# Damage control surgery for perforated diverticulitis with diffuse peritonitis: saves lives and reduces ostomy

**DOI:** 10.1186/s13017-019-0238-1

**Published:** 2019-04-16

**Authors:** Dario Tartaglia, Gianluca Costa, Antonio Camillò, Maurizio Castriconi, Mauro Andreano, Michele Lanza, Pietro Fransvea, Paolo Ruscelli, Massimiliano Rimini, Christian Galatioto, Massimo Chiarugi

**Affiliations:** 1 Emergency Surgery Unit, New Santa Chiara Hospital, University of Pisa, Via Paradisa 2, 56124 Pisa, Italy; 2 Emergency Surgery Unit, Sant’Andrea Teaching Hospital, University Sapienza, Rome, Italy; 3 Emergency Surgery Unit, Ospedale Cardarelli, Naples, Italy; 4 Emergency Surgery Unit, Ospedali Riuniti Teaching Hospital, Polytechnic University of Marche, Ancona, Italy

**Keywords:** Complicated diverticulitis, Damage control surgery, Observational study

## Abstract

**Introduction:**

Over the last decade, damage control surgery (DCS) has been emerging as a feasible alternative for the management of patients with abdominal infection and sepsis. So far, there is no consensus about the role of DCS for acute perforated diverticulitis. In this study, we present the outcome of a multi-institutional series of patients presenting with Hinchey's grade III and IV diverticulitis managed by DCS.

**Methods:**

All the participating centers were tertiary referral hospitals. A total of 34 patients with perforated diverticulitis treated with DCS during the period 2011–2017 were included in the study. During the first laparotomy, a limited resection of the diseased segment was performed followed by lavage and use of negative pressure wound therapy (NPWT). After 24/48 h of resuscitation, patients returned to the operating room for a second look. Mortality, morbidity, and restoration of bowel continuity were the primary outcomes of the study.

**Results:**

There were 15 males (44%) and 19 females (56%) with a mean age of 66.9 years (SD ± 12.7). Mean BMI was 28.42 kg/m^2^ (SD ± 3.33). Thirteen cases (38%) were Wasvary’s modified Hinchey's stage III, and 21 cases (62%) Hinchey's stage IV. Mean Mannheim Peritonitis Index (MPI) was 25.12 (SD ± 6.28). In 22 patients (65%), ASA score was ≥ grade III. Twenty-four patients (71%) had restoration of bowel continuity, while 10 (29%) patients had an end colostomy (Hartmann’s procedure). Three of these patients received a temporary loop ileostomy. One patient had an anastomotic leak. Mortality rate was 12%. Mean length of hospital stay was 21.9 days. At multivariate analysis, male gender (*p* = 0.010) and MPI (*p* = 0.034) correlated with a high percentage of Hartmann’s procedures.

**Conclusion:**

DCS is a feasible procedure for patients with generalized peritonitis secondary to perforated diverticulitis, and it appears to be related to a higher rate of bowel reconstruction. Due to the open abdomen, stay in ICU with prolonged mechanical ventilation is required, but these aggressive measures may be needed by most patients undergoing surgery for perforated diverticulitis, whatever the procedure is done.

## Introduction

Despite the ongoing progress for resuscitation of septic patients, Hartmann’s procedure (HP) is still considered the safest treatment for generalized peritonitis secondary to complicated acute diverticulitis (Hinchey’s classification stages III and IV) [1–3]. However, the optimal surgical treatment for generalized peritonitis due to diverticular perforation remains controversial [4]. Up to half of the patients who underwent HP do not have their stoma reversed as restoration of bowel continuity carries significant morbidity and mortality [5]. To date, the use of resection with primary anastomosis (PA) is a matter of debate; as reported in the literature, PA is substantially equivalent to HP in terms of morbidity, mortality, and length of postoperative stay [6]. PA is often reserved for younger patients with few comorbidities and a lesser degree of peritoneal contamination. Over the last decade, damage control surgery (DCS) has been emerging as a valid alternative to HP and one-stage PA in patients presenting with severe sepsis caused by purulent or fecal peritonitis in acute diverticulitis. Initially described for the treatment of major abdominal injuries, indications for DCS have subsequently been extended to septic shock, abdominal compartment syndrome, and the impossibility to perform a primary closure. This study aimed to describe the effects of damage control surgery on the outcome of patients with purulent and fecal diverticular peritonitis.

## Methods

### Patient selection

The charts of a series of patients who underwent DCS for purulent or fecal diverticular peritonitis in four Italian institutions during the period 2011–2017 were evaluated. Participating centers were tertiary care referral hospitals (Department of Emergency Surgery, Cisanello University Hospital, Pisa, Italy; Department of General Surgery, Cardarelli Hospital, Napoli, Italy; Sant’Andrea University Hospital, Roma, Italy; Emergency Surgery Unit, Polytechnic University of Marche, Ospedali Riuniti Teaching Hospital, Ancona, Italy). Diverticulitis was staged according to Wasvary’s modified Hinchey classification [7]. Inclusion criteria were patients older than 18 years with purulent or fecal peritonitis (Hinchey stage III and IV) secondary to perforated diverticulitis with septic shock and severe grade of peritoneal contamination. The condition of shock was diagnosed according to the parameters of the 3rd International Consensus Definitions for Sepsis and Septic Shock. These are presence of hypotension persisting despite adequate fluid resuscitation and requiring vasopressors with at least one of the following organ failures: kidney failure (creatinine level > 177 mmol/l or urea level > 167 mmol/l or oliguria < 20 ml/h) and pulmonary insufficiency (pO2 < 50 mmHg and pCO2 > 50 mmHg) [8]. All patients underwent clinical assessment and a CT scan before the surgical procedure. Exclusion criteria were age older than 90 years and the presence of poor prognosis-associated comorbidities (es. advanced malignancy) or severe limitation of self-sufficiency (neurologic diseases, caregivers’ dependence) that a priori favored the choice of an end colostomy.

### Surgical procedure

At laparotomy, once the affected bowel segment was resected and the abdominal cavity copiously irrigated with saline solution, the open abdomen (OA) technique was set up by using a negative pressure wound therapy (NPWT, with or without continuous lavage). The decision to restore continuity or create a terminal colostomy was postponed to a second-look operation, 24–48 h later. Occasionally, at the surgeon’s discretion, the OA was maintained following the second-look procedure if the surgical scenario was believed still contaminated and not safe for bowel reconstruction. In such instances, a fascial mesh was placed in order to avoid fascial retraction of the abdominal wall. A defunctioning ileostomy was performed based on the surgeon’s choice when the bowel continuity was re-established. Alternatively, an end colostomy according to Hartmann’s procedure was the choice for those patients in which an intestinal anastomosis was judged unsafe.

### Data analysis and outcome measures

Data analysis included age, gender, Body Mass Index (BMI), American Society of Anesthesiologists (ASA) score, Wasvary’s modified Hinchey's classification stage III and IV rate, severity of peritonitis according to Mannheim Peritonitis Index [9], type of OA technique performed, mean number of looks performed during OA, and modified Bjӧrck classification at II look [10]. Primary outcomes were mortality, overall and specific morbidity according to Clavien and Dindo classification [11], and intestinal anastomosis rate with/without a temporary stoma. Other outcomes included the length of ICU stay, length of total postoperative stay, recurrence of diverticulitis, and late development of an incisional hernia. Follow-up evaluation was based on interval outpatient clinic visit *à la demand* and/or on a telephone interview.

### Statistical analysis

Quantitative data are expressed as mean or median ± standard deviation (SD). The qualitative data were elaborated as frequency and percentage. The Mann-Whitney *U* test was carried out to analyze quantitative variables. Fisher’s exact test was performed to study categorical variables. Univariate analysis of age, male gender, BMI, ASA score > 2, MPI, and Bjӧrck grade at the II look were performed in order the evaluate a statistical difference in patients presenting with end colostomy versus those with an intestinal anastomosis. Any variable with *p* < 0.2 at univariate analysis was entered in a multivariate backward stepwise logistic regression model with Hartmann’s procedure as a dependent variable. The statistical analysis was performed using the IBM SPSS software package, version 21.0 for Mac OsX.

## Results

During the period of study, 34 patients underwent DCS for septic shock secondary to purulent or fecal diverticular peritonitis. Mean age was 66.9 years (SD ± 12.7) (Table 1). There were 15 males (44%) and 19 females (56%). Mean BMI was 28.42 kg/m^2^ (SD ± 3.33). ASA score was > 2 in 22 patients (65%). Based on the severity of the disease, 13 cases (38%) were classified as Wasvary’s modified Hinchey's stage III, and 21 cases (62%) as Hinchey's IV. The mean MPI was 25.12 (SD ± 6.28). A NPWT technique for open abdomen was performed in all patients. In seven cases (21%), there was the need for a third look for the persistence of peritoneal contamination: four of these had an intestinal anastomosis eventually. The modified Björck classification at II look were 1A = 27, 1B = 3, 1C = 2, 2A = 0, 2B = 1, 2C = 1, 3A = 0, 3B = 0, and 4 = 0.

**Table 1:** Univariate analysis

Parameters	Total (*N* = 34)	Anastomosis (*N* = 24)	Hartmann’s (*N* = 10)	*p* value
Male gender	15 (44%)	7 (29%)	8 (80%)	*0.010*
Mean age (± SD)	66.9 (± 12.7)	66.25 (± 11.24)	68.60 (± 16.25)	0.254
BMI (kg/m^2^) (± SD)	28.42 (± 3.33)	27.11 (± 4.09)	30.10 (± 5.89)	0.127
ASA score > II	22 (65%)	16 (67%)	6 (60%)	0.195
MPI (± SD)	25.12 (± 6.28)	23.63 (± 5.87)	28.70 (± 6.03)	*0.034*
Björck grade ≠ 1A at II look	7 (21%)	3 (13%)	4 (40%)	0.157
Hinchey’s classification:				
- Grade III	13 (38%)	11 (46%)	4 (40%)	1
- Grade IV	21 (62%)	13 (54%	6 (60%)	
Type of OA technique adopted: negative pressure wound technique (NPWT)	34 (100%)	24 (100%)	10 (100%)	1
III look	7 (21%)	4 (16.6%)	3 (30%)	0.157
Overall morbidity rate*	14 (41%)	9 (37%)	5 (50%)	0.704
- Grade I	0	0	0	–
- Grade II	6 (18%)	5 (21%)	1 (10%)	–
- Grade IIIa	1 (3%)	1 (4%)	0	–
- Grade IIIb	5 (15%)	3 (13%)	2 (20%)	–
- Grade IV	2 (6%)	0	2 (20%)	–
Cause of reintervention				
- Anastomosis dehiscence	1 (3%)	1 (4%)	–	1
- Laparotomy dehiscence	2 (6%)	1 (4%)	1 (10%)	–
Mortality rate	4 (12%)	1 (4%)	3 (30%)	0.066
Mean length of hospital stay (± SD) (days)	21.9 (± 16.24)	22.45 ± 17.74	20.5 ± 11.72	0.752

*According to the Clavien and Dindo classification

Italics indicate statistical significance

In 24 cases (71%), the intestinal continuity was restored, and in 3 of these (12%), a protective loop ileostomy was performed. Ten patients had an end colostomy as a result of the DCS. The overall morbidity rate was 41% (14/34). According to the Clavien and Dindo classification, there were 0 grade I (0%), 6 grade II (18%), 6 grade III (18%), and 2 grade IV (6%). In three cases (9%), a re-operation was needed: in one case for an anastomotic leak and two cases for dehiscence of the laparotomy wound. In the patient with the bowel leak, the anastomosis was dismantled, and an end colostomy was warranted.

The mean ICU length was 13.98 days (SD ± 13.47), and the mean postoperative stay was 21.9 days (SD ± 16.24). Four deaths (12%) were recorded: three patients following a Hartmann’s procedure and one after bowel anastomosis (*p* = 0.067; odds ratio 3.067 [95% CI 0.555–16.935]). All deaths were related to the multiorgan failure. Mean follow-up was 47 months (SD ± 35.67); all patients with loop ileostomy underwent closure of ileostomy. None of the patients presenting with an end colostomy had a Hartmann’s reversal. So far, the incisional hernia developed in 8 out 30 patients (27%) seen on our patient visit. No recurrence of diverticulitis episodes occurred.

At univariate analysis, male gender (*p* = 0.010) and MPI (*p* = 0.034) correlate with a high percentage of Hartmann’s procedures (Table 1). These parameters result at multivariate analysis as predictive factors for an end colostomy creation (Table 2).

**Table 2:** Multivariate analysis

		B	E.S.	Wald	df	Sig.	Exp (B)	95% CI	
								Inferior	Superior
Step 1	Male gender	3.141	1.366	5.286	1	0.021	23.136	1.590	336.734
	BMI	0.162	0.156	1.080	1	0.299	1.176	0.866	1.596
	MPI	0.141	0.105	1.802	1	0.179	1.151	0.937	1.414
	Björck grade	1.614	1.527	1.117	1	0.291	5.023	0.252	100.141
	ASA > 2	.103	1.582	0.004	1	0.948	1.109	0.050	24.647
	Constant	− 1.178	5.832	3.674	1	0.055	0.000		
Step 4	Male gender	2.700	1.050	6.613	1	0.010	14.881	1.901	116.509
	MPI	0.70	0.077	4.931	1	0.026	1.185	1.020	1.377
	Constant	− 6.750	2.336	8.349	1	0.004	0.001		

## Discussion

Perforated diverticulitis with generalized peritonitis requires immediate surgical intervention. A high percentage of these patients develop severe septic shock with multiorgan dysfunction [12]. Over the last decade, damage control surgery has been emerging as possible alternative management of patients with abdominal infection and sepsis. So far, there is no consensus about the role of DCS in acute perforated diverticulitis. Hartmann’s procedure remains the therapy of choice in this critical situation in many medical institutions. Indeed, in those patients with pre-existing life-threatening comorbidities, it could be not reasonable to go for a primary anastomosis. However, several studies suggest that the mortality rate is the same for the Hartmann procedure and primary anastomosis [4, 13, 14].

In a recent randomized multicenter study, Bridoux et al. demonstrate on 102 patients with diverticular peritonitis that overall mortality did not differ significantly between HP (7.7%) and PA (4%) and that morbidity for both resection and stoma reversal operations was comparable (39% HP vs. 44% PA). Furthermore, they found that at 18 months, 96% of PA patients with loop ileostomy and 65% of HP patients had a stoma reversal. Thus, they concluded that PA with diverting ileostomy is preferable respect to HP in patients with diverticular peritonitis [6].

Moreover, it has been demonstrated that Hartmann’s reversal with the restoration of the bowel continuity is associated with a significant high rate of morbidity (60%), anastomotic leak (30%), and mortality (up to 15%) [15]. Colostomy complications often require additional surgical procedures with increased morbidity. Many patients (20 to 50%) may even never have their colostomy reversed because of severe perioperative risks [16–19].

Primary anastomosis has been widely accepted as an alternative to HP with comparable mortality and a low risk of anastomotic leakage [20–23]. The procedure is often reserved for patients with low-grade comorbidities and less peritoneal contamination [16, 19, 23]. However, a scenario of fecal or purulent peritonitis caused by acute diverticulitis and the presence of septic shock may keep the creation of an anastomosis not feasible or unsafe. In this setting, DCS matches the purpose of obtaining the control of the septic source with the purpose of offering a bowel reconstruction under better abdominal conditions [23].

The recent World Society of Emergency Surgery guidelines about the management of intra-abdominal sepsis state that there is insufficient evidence to advocate for DCS as a general strategy in patients with secondary peritonitis (Recommendation 1 C). On the other hand, even though with a low grade of recommendation (Recommendation 2 C), the same guidelines suggest that DCS may be an option in selected significantly physiologically deranged patients with ongoing sepsis [24].

Only a few case series focusing on DCS for perforated diverticulitis are so far reported. Perathoner et al. were the first to describe the combination of emergency laparotomy with the application of abdominal vacuum-assisted closure (V.A.C.®) to treat patients with generalized peritonitis in 2010 [25]*.* They analyzed 15 patients who underwent DCS with lavage, limited resection of the diseased colonic segment, VAC, and second-look operation; 9 of them had intestinal continuity restored during a second-look operation, with 1 patient developing anastomotic leakage. More recently, Sohn et al. reported on 58 patients undergone DCS for Hinchey III (81%) and Hinchey IV (19%) acute diverticulitis; they recorded a secondary bowel restoration rate of 83% with a 24% of loop ileostomy rate. Ten percent of their patients experienced an anastomotic leak that eventually required the creation of a stoma. At the end of the hospital stay, they scored a 50% rate of patients without a stoma [23]. Kafka-Ritsh et al. reported a series of 51 patients with diverticular peritonitis where the rate of bowel restoration at the second-look operation was 76% with an 11% rate of loop ileostomy. Their fistula rate was 13%. [26].

A careful selection of patients that may have benefited from DCS is mandatory in order to avoid overtreatment. It is evident that OA with multiple steps surgery is a risky and costly procedure, and for these reasons, it should not be carried out to merely restore bowel continuity. The primary target of the procedure should be to save lives while to avoid a stoma comes as a reasonable consequence of the first. In our study, 68% of all patients were discharged with a colonic anastomosis. The mortality rate was 12%, slightly higher than data reported in the literature (9–9.8%) [23, 26]. In our cohort, neither fistula formation nor bleeding, that represents an important complication of negative pressure therapy in OA, were observed.

Moreover, a dynamic fascial closure and early removal of the NPWT system allowed for primary closure of the abdominal wall in all patients, although 27% of patients developed an incisional hernia in the follow-up. Anastomotic leak occurred in one patient (3%), and an end colostomy managed it at re-operation. At the multivariate analysis, male gender and MPI turned out to be the predictive factors for an end colostomy creation at the II look.

We are fully aware of several limitations affecting the present study. Firstly, it has a relatively low number of enrolled patients, and it cannot be excluded a bias in patients’ selection. Moreover, the study is retrospective, and it encompasses a quite large period. Finally, reliable criteria backing the decision to restore bowel continuity or to bail out with an end colostomy at the time of second look are lacking.

## Conclusions

Damage control strategy represents a feasible strategy for patients with generalized peritonitis secondary to perforated diverticulitis. This surgical approach seems to offer a high rate of bowel reconstruction with less stoma, without increasing mortality and morbidity. It requires a stay in ICU with prolonged mechanical ventilation, a hefty price that however these patients should have paid to septic shock independently from the type of surgery. Further randomized, controlled, and comparative studies are needed to achieve significant conclusions.

## Abbreviations

ASA: American Society of Anesthesiologists
BMI: Body mass index
DCS: Damage control surgery
HP: Hartmann’s procedure
ICU: Intensive care unit
NPWT: Negative pressure wound therapy
OA: Open abdomen
PA: Primary anastomosis
